# Marine Compounds and Cancer: The First Two Decades of XXI Century

**DOI:** 10.3390/md18010020

**Published:** 2019-12-26

**Authors:** Sergey A. Dyshlovoy, Friedemann Honecker

**Affiliations:** 1 Laboratory of Pharmacology, A.V. Zhirmunski National Scientific Center of Marine Biology, Far Eastern Branch, Russian Academy of Sciences, 690041 Vladivostok, Russia; 2 Department of Oncology, Hematology and Bone Marrow Transplantation with Section Pneumology, Hubertus Wald-Tumorzentrum, University Medical Center Hamburg-Eppendorf, 20251 Hamburg, Germany; 3 Laboratory of Biologically Active Compounds, Department of Bioorganic Chemistry and Biotechnology, School of Natural Sciences, Far Eastern Federal University, 690091 Vladivostok, Russia; 4 Martini-Klinik, Prostate Cancer Center, University Hospital Hamburg-Eppendorf, 20251 Hamburg, Germany; 5 Tumor and Breast Center ZeTuP St. Gallen, 9000 St. Gallen, Switzerland

In 2019, the scientific and medical community celebrated the 50th anniversary of the introduction of the very first marine-derived drug, Cytarabine, into clinics. Cytarabine (aka Ara-C, Cytosar-U^®^) was first isolated from a marine sponge and is known to kill cancer cells by blocking DNA polymerase function [1]. In 1969, the FDA approved the drug for the treatment of leukemia. This marine drug, which still belongs to the mainstay of leukemia therapy, and by now has probably saved many thousands of lives, made its way into clinics in less than 20 years after the original prototype molecules, namely spongothymidine and spongouridine, were reported by Bergmann and Feeney in 1951 [2]. Some years later, in 1976, another marine drug, Vidarabine (Ara-A, Vira-A^®^), was approved for the treatment of *Herpes simplex* virus. However, after that, the clinical development of marine-derived drugs was less successful [1], and for another almost 40 years, no other compounds were approved by drug authorities. Moreover, the rapid development of methods of high-throughput screening, and especially computational approaches to rational drug design, made some scientists believe that the search for new bioactive molecules from natural sources was an activity of the past. Thus, by the end of the last century, a part of the scientific community was rather skeptic on whether new natural products and, in particular, marine natural products, still harbored the potential to make new drugs. However, having passed this transition period, the situation has definitely changed, and at the beginning of the 21st century, marine drugs entered a time of renaissance [3,4]. Nowadays, while many terrestrial animals and plants are already well investigated, marine inhabitants have become the main source of new chemical compounds. Moreover, due to the specific (and often extreme) environmental conditions, marine inhabitants often possess particular biochemistry which results in unique secondary metabolites. These organisms are mainly produced by marine invertebrates like sponges and tunicates (*Ascidia*), and marine fungi and bacteria, the latter often being the true producers of small bioactive molecules of interest.

Currently, the potential for marine natural products as drug candidates has been recognized all over the world, and the field is constantly growing and developing. Additionally, the development of new chemical and physicochemical approaches and tools has led to the isolation and structure elucidation of novel minor marine secondary metabolites, which could not be isolated/detected in the past. The number of structures isolated each year has almost doubled over the past 20 years. This is illustrated by the fact that according to the report of John Faulkner, 869 new structures were isolated from marine organisms during the year 2000 [5]. A decade later, in 2010, the number had risen to 1003 substances per year, as reported by John Blunt et al. [6], and a very recent report by Anthony Carroll et al. described 1490 new molecules isolated in the year 2017 [7]. Moreover, the development of new and improved organic synthesis methods made possible the synthesis of promising active compounds in the amounts required for further preclinical and clinical studies.

Tremendous progress in the clinical development of marine-derived drugs has been achieved over the past 20 years. For example, during this period, six out of nine currently used drugs of marine origin have been approved by their corresponding authorities [8]. Focusing specifically on anti-cancer drugs, two more antineoplastic agents, Plitidepsin [9] and Polatuzumab vedotin [10], have been approved since we reviewed this topic in 2017 [11]. Hence, the full list of the marine-derived drugs used for cancer treatment at the end of 2019 included:

(1) Spongian nucleoside **Cytarabine** (Ara-C, Cytosar-U^®^ (*Pfizer*), see above);
(2) Spongian macrolide **Eribulin mesylate** (E7389, Halaven^®^ (*Eisai Inc.*), first approved in 2010 for the treatment of metastatic breast cancer; mechanism of action—irreversible mitotic blockade);
(3) **Brentuximab vedotin**—an antibody-drug conjugate (ADC) of the CD30-specific monoclonal antibody brentuximab with the antimitotically active monomethyl auristatin E (MMAE), which is a synthetic analog of dolastatin-10, produced by cyanobacteria symbiotic to sea hare *Dolabella auricularia* (SGN-35, Adcetris^®^ (*Seattle Genetics*), which was first approved in 2011 for the treatment of anaplastic large T-cell systemic malignant lymphomas, and Hodgkin’s lymphomas; mechanism of action—binding to CD30 (antibody) and inhibition of tubulin polymerization (MMAE));
(4) Ascidian alkaloid **Trabectidine** (ET-743, Yondelis^®^ (*PharmaMar*), first approved in 2015 for the treatment of soft tissue sarcoma and ovarian cancer, mechanism of action—binding to the minor groove of DNA);
(5) The ascidian depsipeptide **Plitidepsin** (dehydrodidemnin B, Aplidin^®^ (*PharmaMar*), first approved in 2018 for the treatment of leukemia, lymphoma, and multiple myeloma, mechanism of action—induction of oxidative stress, binding to eEF1A2). Note: Aplidin^®^ is currently approved by the Australian Regulatory Agency for use in Australia only;
(6) **Polatuzumab vedotin**—another antibody-drug conjugate which consists of a CD76b-targeting antibody and MMAE (DCDS-4501A, Polivy^TM^ (*Genetech, Roche*), and was first approved in 2019 for the treatment of chronic lymphocytic leukemia, B-cell lymphomas, non-Hodgkin lymphomas, mechanism of action—binding to CD76b (antibody) and inhibition of tubulin polymerization (MMAE)).

By the end of 2019, there were an additional 28 drug candidates in different stages of clinical trials. The majority of them (24 substances, 85%) are undergoing clinical trials as anti-cancer drugs. Interestingly, 20 out of 24 molecules (83%) are antibody-drug conjugates (ADC) and therefore consist of a small cytotoxic “warhead” molecule and a specific antibody targeting different cellular membrane proteins.

Currently, five compounds are undergoing testing in phase III clinical trials and are at the final step before approval can be obtained. Apart from the well-known Tetrodotoxin (Halneuron^TM^, *Wex Pharmaceutical Inc*.), which is being developed as a painkiller for chronic, severe to moderate cancer-related pain, the other four candidates are all investigated as potential chemotherapeutic agents. In more detail, these substances are the nectin-4 targeting ADC Enfortumab Vedotin (ASG-22ME, *Seattle Genetics*) which is being tested in several urogenital tumors; the synthetic analog of the fungian diketopiperazine Plinabulin (NPI-2358, *BeyondSpring Pharmaceuticals Inc.*), which is able to inhibit tubulin polymerization and is tested in brain tumors and non-small cell lung cancer (NSCLC); the DNA minor groove binder Lurbinectedin (PM01183, Zepsyre^®^, *Pharmamar*), a synthetic analog of trabectedin which currently is tested in small cell lung cancer (SCLC), ovarian and breast cancers; and the bacterial proteasome inhibitor β-lactone-γ-lactam Marizomib (NPI-0052, Salinosporamide A, *Triphase*), which has been examined in melanomas, lymphomas, NSCLC, pancreatic cancers, as well as multiple myelomas.

On the other hand, most of the molecules undergoing phase I and phase II trials are monomethyl auristatin E or F (MMAE or MMAF) conjugates with different antibodies [8]. It seems a safe bet that we may expect several MMAE/MMAF based drugs to make it into clinics in the next decade. Excitingly, there are around 2000 marine-derived molecules that show interesting *in vivo* biological activity, and more than 10,000 compounds isolated from marine organisms for which *in vitro* activity has been described [12].

Drug development is a very dynamic field, as new and old drug candidates regularly enter phase I trials for different cancers, go on to the next trial phases, or fail. Therefore, this information should be regularly revised. For those who are interested in the most up-to-date status of different drug candidates, we recommend visiting the Marine Pharmacology web-site (https://www.midwestern.edu/departments/marinepharmacology.xml) ran and regularly updated by former Editor-in-Chief of Marine drugs Prof. Alejandro M. S. Mayer (Midwestern University, IL, USA), and his team. Vital information can also be obtained from www.clinicaltrials.gov, www.accessdata.fda.gov, or directly from the web-pages of pharmaceutical companies involved in marine drug development.

The main obstacle for novel marine-derived molecules on their way to becoming clinically useful drugs (i.e., anti-cancer drugs) is the generation of larger amounts of the active substance. At the beginning of the era of clinical use of marine drugs, naturally harvested or maricultured organisms were used as the main source of an active compound. As an example, the first hundreds of milligrams of halichondrin B and trabectedin were produced from marine invertebrates cultured in marine farms by the US National Cancer Institute [13], and PharmaMar [14], without which preclinical and clinical trials and further development of the meanwhile approved drugs Halaven^®^ and Yondelis^®^ would not have been possible. Nowadays, interesting compounds are largely produced by chemical synthesis or by modifying natural compounds that are mass-produced by microorganisms, either in wild-type or genetically modified cultures. However, macromolecules, such as marine polysaccharides and their derivatives (mainly used as food, as food additives, or in the cosmetic industry), are isolated and purified from natural sources like marine alga. There are some exceptions to this rule, e.g., several small molecules possessing anti-cancer activity isolated directly from the sponge *Aplysina aerophoba* marketed by BromMarin [15]. 

*Marine Drugs* (ISSN 1660-3397) is a leading journal on the research, development, and production of biologically and therapeutically active compounds from the sea. To document the dynamic field of marine anti-cancer pharmacology, the Topical Collection “Marine Compounds and Cancer” (http://www.mdpi.com/journal/marinedrugs/special_issues/marine-compounds-cancer) was started three years ago [11]. This Topical Collection covers the whole scope from agents with cancer-preventive activity to novel and previously characterized compounds with anti-cancer activity, both in vitro and in vivo, and the latest status of clinical development from drug trials. Of note, compounds possessing pro-carcinogenic activity or mediating cancer cell survival are also within the scope of the collection. Owing to the importance of trial execution, special attention is given to current shortfalls and possible strategies to overcome obstacles in the area of marine anti-cancer drug development.

These are exciting times for scientists and physicians involved in the investigation and development of marine drugs! We invite the authors to share with us and all our readers your latest and forward-looking discoveries!

Dr. Sergey A. Dyshlovoy and Dr. Friedemann Honecker, Guest Editors, Topical collection “Marine Compounds and Cancer”.
